# Intramedullary fixation of distal fibular fractures: a systematic review of clinical and functional outcomes

**DOI:** 10.1007/s10195-014-0320-0

**Published:** 2014-10-11

**Authors:** Sameer Jain, Benjamin A. Haughton, Christopher Brew

**Affiliations:** Bradford Royal Infirmary, Duckworth Lane, Bradford, West Yorkshire BD9 6RJ UK

**Keywords:** Fibular, Ankle, Fracture, Intramedullary

## Abstract

**Background:**

Ankle fractures are extremely common and represent nearly one quarter of all lower-limb fractures. In the majority of patients, fractures involve the distal fibula. The current standard in treating unstable fractures is through open reduction and internal fixation (ORIF) with plates and screws. Due to concerns with potentially devastating wound complications, minimally invasive strategies such as intramedullary fixation have been introduced. This systematic review was performed to evaluate the clinical and functional outcomes of intramedullary fixation of distal fibular fractures using either compression screws or nails.

**Materials and methods:**

Numerous databases (MEDLINE, PubMed, Embase, Google Scholar) were searched, 17 studies consisting of 1,008 patients with distal fibular fractures treated with intramedullary fixation were found.

**Results:**

Mean rate of union was 98.5 %, with functional outcome reported as being good or excellent in up to 91.3 % of patients. Regarding unlocked intramedullary nailing, the mean rate of union was 100 %, with up to 92 % of patients reporting good or excellent functional outcomes. Considering locked intramedullary nailing, the mean rate of union was 98 %, with the majority of patients reporting good or excellent functional outcomes. The mean complication rate across studies was 10.3 %, with issues such as implant-related problems requiring metalwork removal, fibular shortening and metalwork failure predominating.

**Conclusion:**

Overall, intramedullary fixation of unstable distal fibular fractures can give excellent results that are comparable with modern plating techniques. However, as yet, there is unconvincing evidence that it is superior to standard techniques with regards to clinical and functional outcome.

**Level of evidence:**

Level IV evidence.

## Introduction

Ankle fractures were first described by Sir Percival Pott in 1768 and are one of the most common skeletal injuries seen in clinical practice [1]. It has been estimated that they comprise 9 % of all fractures and up to 22.6 % of all lower-limb fractures in the UK population [2, 3]. Their prevalence is rising as a consequence of osteoporosis in an increasing elderly population [4]. An epidemiological study of 1,500 ankle fractures revealed that isolated distal fibular or lateral malleolus fractures occurred in two thirds of patients, whilst bimalleolar fractures occurred in a quarter and trimalleolar fractures in the remaining 7 % [5].

The two most universally accepted classification schemes are the Danis-Weber and Lauge-Hansen systems [6–8]. Whilst both allow clinicians to define and communicate the fracture pattern, managing these injuries is primarily based on an assessment of stability, which incorporates the amount of displacement, presence of medial injury and associated talar shift [9, 10]. The treatment aims are to obtain reduction of displaced fractures, maintain anatomic alignment of the ankle mortise and achieve bony union. The closed treatment of stable fractures routinely involves a short period of casting and is usually successfully followed by a progressive return to weight bearing and physiotherapy [11, 12]. Unstable fractures with disruption of the mortise require open reduction and internal fixation (ORIF). Initially, this involves closed reduction and temporary stabilisation by casting or external fixation. ORIF can be performed safely once soft tissue swelling has settled in order to reduce the risk of wound complications [13]. Most commonly, ORIF is performed using Arbeitsgemeinschaft für Osteosynthesefragen (AO) principles through the use of compression screws and a neutralisation plate [14]. Precontoured locking plates have been introduced to allow improved fixation in osteoporotic bone [15]. However, lateral plating can lead to complications such as wound breakdown and infection due to the poor skin envelope that surrounds the distal fibula [16]. Furthermore, patients often complain of prominent hardware that require subsequent removal [17, 18]. A recent systematic review of 1,822 ankle fractures treated with ORIF revealed that approximately one fifth of optimally reduced fractures had unsatisfactory results with regards to functional outcome, subjective outcome and radiographic evaluation [19].

Intramedullary fixation includes the use of both compression screws and intramedullary nailing (IMN). As mini-incision techniques are used, these techniques may benefit patients with compromised skin by reducing the risk of wound complications. In addition, soft-tissue swelling may not present a contraindication to early fixation, potentially allowing earlier surgery and thus earlier discharge from hospital. However, a formal assessment of the overall outcomes of patients treated with these methods has never been made. Therefore, the aim of this systematic review was to evaluate the clinical and functional results of patients with distal fibular fractures treated with intramedullary devices.

## Materials and methods

**Fig. 1:**
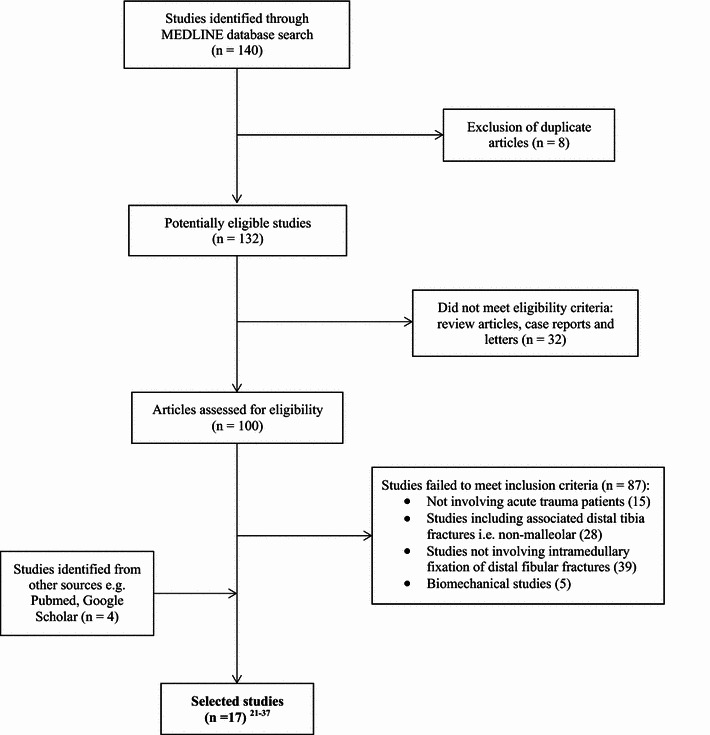
Results of literature search and application of eligibility criteria

Preferred Reporting Items for Systematic Reviews and Meta-Analyses (PRISMA) guidelines were consulted throughout this review [20]. A comprehensive literature search was performed on 24 November 2013 using Ovid at MEDLINE (1946–2013). Search terms using Boolean operators were fibular fracture OR ankle injuries/or ankle fracture AND bone nails/or fracture fixation, intramedullary/or nailing and were limited to the English language and human studies. In combination, these search terms resulted in a total of 140 articles. A review of abstracts was then performed based on the following inclusion criteria: patients with a distal fibular fracture treated with an intramedullary device; and studies reporting union rates, functional outcome and complications. Exclusion criteria included duplicate results, studies not involving intramedullary fixation for fibular fractures, studies involving fixation of associated distal tibial fractures (i.e. nonmalleolar fractures), biomechanical studies, case reports, review articles, comments and letters. If the abstracts did not reveal the desired information, the complete articles were obtained and filtered appropriately. After application of eligibility criteria, this search revealed 13 suitable studies [21–33]. A further search was repeated on the PubMed and Embase and revealed one further study [34]. A search on Google Scholar revealed another study and two abstracts presented at recent national meetings [35–37]. A search for the terms ankle was also performed on the Cochrane Database of Systematic Reviews. References were also studied in each of the retrieved papers, but these processes revealed no further studies. A flow diagram is presented in Fig. 1 showing results of the literature search by two researchers (SJ, BAH) in order to prevent any important omissions. Any disagreements regarding study eligibility were settled through discussion.

## Results

In total, 17 studies were selected for review with regards to both clinical and functional outcome [21–37]. Three studies involve intramedullary screw fixation [24, 25, 31], and 14 studies involved IMN [21–23, 26–30, 32–37] of distal fibular fractures. Due to the inherent differences in these fixation methods, these techniques were analysed separately. Furthermore, studies involving IMN were subdivided into those using unlocked and those using locked nails. Due to the heterogeneity of the study population and fixation devices, data synthesis was not possible for an accurate meta-analysis.

### Intramedullary screw fixation

Three articles of level IV scientific evidence [38] were reviewed (Table 1) [24, 25, 31]. These included 91 patients with a mean age of 37.9 (range 37–39.5) years [24, 25, 31]. There were 45 men and 46 women, with a mean follow-up of 12.1 (range 8–15.9) months [24, 25, 31]. The studies used different classification systems, but in total, there were 31 lateral malleolar fractures, 48 bimalleolar fractures and 12 trimalleolar fractures [24, 25, 31]. All were either Weber B or low Weber C type fractures. A variety of techniques and implant devices were used. Two studies [24, 31] incorporated open reduction of the fibular fracture into their surgical technique, whilst one study [25] used a closed reduction technique throughout their series. Two studies used a 4.2-mm, fully threaded, self-tapping Woodruff screw (Zimmer, Warsaw, IN, USA) inserted in compression mode for fixation [24, 25]. The other study used a cannulated, variable-pitch, headless compression screw for fixation (Acutrak plus compression screw; Acumed Inc., Beaverton, OR, USA) [31]. Various methods of fixation of associated medial malleolus fractures were used throughout, e.g. screws, wires, plates.

**Table 1:** Results of intramedullary screw fixation

References	Patients	Age (years)	Male:female	Follow-up (months)	Technique/implant	Union rate (%)	Functional outcome	Complications	Complication rate (%)
Bankston et al. [24]	44	39.5	19:25	8	Open reduction, 4.2-mm fully threaded screw	100	Time to weight-bearing: 7.2 weeks	Wound leakage 2	9
								Malunion 1	
								Metalwork removal 1	
Ray et al. [25]	24	37	13:11	15.9	Closed reduction, 4.2-mm fully threaded screw	95.5	Time to weight bearing: 6.8 weeks, 84.2 % good or excellent subjective outcome	Wound leakage 1	12.5
								Nonunion 1	
								Malunion 1	
Lee et al. [31]	23	37.4	13:10	12.4	Open reduction, headless variable-pitch compression screw	100	91.3 % good or excellent subjective outcome	Superficial infection 1	4.3

All values represented as mean unless otherwise stated

Mean union rate was 98.5 % (range 95.5–100 %) [24, 25, 31]. Functional outcome was assessed subjectively through telephone-based questionnaires and was found to be good or excellent in 84.2–91.3 % of patients [25, 31]. In addition, mean time to weight bearing was reported in two studies and occurred at 6.8–7.2 weeks [24, 25]. The mean complication rate across studies was 8.6 % (range 4.3–12.5 %), with reported complications being wound leakage in three cases, malunion in two, nonunion in one, metalwork prominence requiring removal in one and superficial wound infection requiring a course of antibiotics orally in one [24, 25, 31].

### Unlocked intramedullary nail fixation

Six articles of level III [23, 30, 34] or IV [21, 22, 35] evidence were retrieved (Table 2) [21–23, 30, 34, 35]. One study [22] contained a cohort of patients that was evaluated in a previous study [21]. In order to avoid data replication, only the most recent cohort from the second study [22] was included. Overall, 290 patients with a mean age of 51.6 (range 37–74) years were included [21–23, 30, 34]. One study did not report the mean age of their study population [35]. There were 95 male and 120 female patients, with a mean follow-up of 27.1 (range 12–36) months [22, 23, 30, 34, 35]. One study did not report the follow-up time or male–female ratio [21]. Again, different classification systems were used. Overall, their were 85 supination–external rotation injuries, four supination–adduction injuries and 11 pronation–external rotation injuries [21, 23]. In addition, 18 isolated lateral malleolus, 111 bimalleolar and eight trimalleolar fractures were reported [30, 34, 35]. One study did not classify fractures [22]. Both open and closed reduction manoeuvres were used prior to nail insertion. As before, associated medial malleolus fractures were treated with a variety of techniques. A variety of implants were used throughout the series, including the Inyo nail (Richards Medical Company, Memphis, TN, USA) in 150 cases, Rush rods (Berivon, Meridian, MS, USA) in 25 cases, the Epiphysa fibular nail (manufacturer unspecified) in 45 cases and Knowles pins (Zimmer) in 70 cases [21–23, 30, 34, 35].

**Table 2:** Results of unlocked intramedullary nail fixation

References	Patients	Age (years)	Male: female	Follow-up (months)	Implant	Union rate (%)	Functional outcome	Complications	Complication rate (%)
McLennan and Ungersma [21]	75	37	NR	NR	Inyo nail	100	85 % good result	Malunion 5	16
								Nail migration 7	
McLennan and Ungersma [22]	75	42	27:48	24 (minimum)	Inyo nail	100	Time to weight-bearing: 4 weeks 90 % good result	Complex regional pain syndrome 4	10.6
								Osteoarthritis 2	
								Symptomatic nail removal 2	
Pritchett [23]	25	74	10:15	36	Rush rods	100	Time to weight-bearing: 6 weeks 60 % good result	Symptomatic nail removal 3	12
Francois et al. [35]	45	NR	23:22	12 (minimum)	Epiphysa fibular nail	100	82.2 % satisfactory or excellent outcome	Symptomatic nail removal 16	Incompletely reported
								Incompletely reported	
Lee et al. [34]	45	62.7	20:25	34.5	Knowles pin	100	Baird and Jackson ankle score 94.2 (good)	None	0
Lee et al. [30]	25 (open fractures)	40.1	15:10	29	Knowles pin	100	92 % good or excellent outcome	Symptomatic nail removal 1	4

All values represented as mean unless otherwise stated

*NR* not reported

The mean observed union rate was 100 %, with not a single reported occurrence of nonunion of the distal fibular fracture [21–23, 30, 34, 35]. The methods of reporting functional outcome varied, but 60–92 % of patients subjectively reported good-to-excellent outcomes [21–23, 30, 34, 35]. Only one study used a validated ankle scoring system and obtained a mean Baird and Jackson score [39] of 94.2, i.e. good outcome. In addition, the time to weight bearing was reported in two studies as 4–6 weeks [22, 23]. Complications were clearly reported in all but one study [35]. These included symptomatic hardware requiring removal in 22 cases, nail migration in seven, malunion in five, complex regional pain syndrome in four and posttraumatic osteoarthritis in two [21–23, 30, 34, 35]. Overall mean complication rate was 8.5 % (range 0–16 %) [21–23, 30, 34, 35].

### Locked intramedullary nail fixation

Eight articles of level I [37], III [36] and IV [26–29, 32, 33] evidence were identified for analysis (Table 3) [26–29, 32, 33, 36, 37]. These include two recent abstracts presented at recent national meetings [36, 37]. Overall, these studies include 627 patients with a mean age of 60.7 (range 36.3–79) years [27–29, 32, 33, 36, 37]. One study did not present mean patient age but instead gave a range of 19–70 years [26]. There were 227 male and 382 female patients, with a mean follow-up of 26.7 (range 5.4–72) months [26–29, 32, 33, 36, 37]. One study did not report their male to female ratio [36]. As before, different classification systems were used. In total, there were 151 Weber B fractures and 113 Weber C fractures [26, 28, 29, 36]. In addition, 25 supination–external rotation, 11 pronation–abduction and 13 pronation–external rotation injuries were reported [27]. Also, there were 24 isolated lateral malleolus, 68 bimalleolar, 35 trimalleolar and two pilon fractures reported [32, 33]. One abstract did not provide detail with respect to fracture classification [37]. Again, both open and closed reduction manoeuvres were used prior to nail insertion, and associated medial malleolus fractures were treated through a variety of techniques. Implants used were the XS nail (Intraplant, Endocare, Germany) in 194 cases, the ANK nail (manufacturer unspecified) in 177 cases, the Acumed fibular nail (Hillsboro, Oregon, USA) in 105 cases, the SST locked nail (Biomet, Warsaw, IN, USA) in 33 cases and an unspecified device in 118 cases [26–29, 32, 33, 36, 37].

**Table 3:** Results of locked intramedullary nail fixation

References	Patients	Age (years)	Male:female	Follow-up (months)	Implant	Union rate (%)	Functional outcome	Complications	Complication rate (%)
Kara et al. [26]	128	19–70 (range)	72:56	37.2	ANK nail	100	74 % good; 22 % fair (subjective)	Posttraumatic osteoarthritis 3	10.9
								Fibular shortening 11	
Kabukcuoglu et al. [27]	49	36.3	29:20	39	ANK nail	NR	Baird and Jackson score; 59.2 % excellent; 24.5 % good	Posttraumatic osteoarthritis 3	20.4
								Fibular shortening 4	
								Malunion 3	
Ramasamy and Sherry [28]	9	67.2	2:7	25.9	SST locked nail	88.9	OMS mean not reported; 87.5 % excellent; 12.5 % good	Posttraumatic osteoarthritis 2	33.3
								Mechanical failure 1	
Gehr et al. [29]	194	49.7	78:116	15	XS nail	99.4	OMS mean not reported; 58.6 % excellent; 33.3 % good	Haematoma 2	8.6
								Infection 1	
								Wound breakdown 2	
								Refracture 1	
								Nonunion 1	
								Metalwork problems requiring further surgery 7	
Rajeev et al. [32]	24	79	2:22	7	SST locked nail	100	OMS 58.125 (mean); 37.5 % good; 62.5 % fair	None	0
Bugler et al. [33]	105	64.8	19:86	72	Acumed fibular nail	100	OMS 65 (mean); 46 % good; 40 % fair. AAOS F and A score 83; SF-12 score 46	Mechanical failure 7	22.8
								Infection 5	
								Metalwork problems requiring further surgery 4	
								Screw impingement 7	
								Myocardial infarction 1	
Tawari et al. [36] (abstract)	18	53.6	NR	5.4	NR	100	Time to weight bearing: 8.4 weeks	None	0
Bugler et al [37] (abstract, prospective, randomised controlled trial)	100 patients total	74	25:75	12	Group 1: acumed fibular nail	NR	OMS no significant difference	Group 1	0
								None	
								Group 2	Insufficient detail
					Group 2: standard plating				
								Infection 6	
								Wound breakdown 2	

All values represented as mean unless otherwise stated

*NR* not reported, *OMS* Olerud and Molander score, *AAOSF&A* American Academy of Orthopaedic Surgeons Foot & Ankle score, *SF-12* Short-Form 12, General Health Survey

Mean union rate across the series was 98 % (range 88.9–100 %) [26–29, 32, 33, 36, 37]. Two studies did not report their union rates [27, 37]. Functional outcome was again measured through a variety of assessment tools; most commonly used was the Olerud and Molander score (OMS) [40], used in five studies [28, 29, 32, 33, 37]. Mean OMS was reported in two studies, giving an overall mean score of 61.6, i.e. good’ [32, 33]. Across four studies, OMS was excellent in 36.5 %, good in 32.3 %, fair in 25.6 % and poor in the remaining 5.6 % [28, 29, 32, 33]. In the only level I study, the mean OMS did not significantly differ between patients treated with locked IMN and patients treated using conventional ORIF techniques [37]. Complications were generally well reported throughout and included fibular shortening in 13 cases, metalwork problems needing further surgery in 11, posttraumatic osteoarthritis in eight, mechanical failure in eight, locking screw impingement in seven, infection in six, wound breakdown requiring skin grafting in four, malunion in three, haematoma in one, refracture in one and nonunion in one [26–29, 32, 33, 36, 37]. One systemic complication was reported in the form of a postoperative myocardial infarction [33]. The overall mean complication rate was 12 % (range 0–33.3 %) [26–29, 32, 33, 36, 37].

## Discussion

Intramedullary fixation is a well-established technique for managing long-bone fractures. Standard AO plating of distal fibular fractures achieves acceptable and consistent union rates but has been associated with wound infection, wound breakdown and hardware prominence, with reported complication rates of up to 30 % [41–43]. Due to the mini-incision technique and low-profile implants associated with intramedullary fixation of distal fibular fractures, there is a theoretical reduction in the risk of patients developing wound complications and soft-tissue irritation due to hardware prominence. The purpose of this systematic review was to evaluate the results of intramedullary fixation with regards union, functional outcome and complications.

Earlier studies evaluated the outcome of distal fibular fractures treated with intramedullary screws [24, 25, 31]. Bankston et al. [24] used open reduction techniques and inserted 4.2-mm fully threaded screws in compression mode. Cerclage wires were used at the surgeons’ discretion for improved stability at the fracture site. Ray et al. [25] specified that the fracture pattern must be transverse and short oblique or minimally comminuted, otherwise it is not possible to maintain fibular length with intramedullary screws. They used closed reduction techniques under image guidance with occasional percutaneous use of a towel clip. An advantage of this method is that patients can be treated on an outpatient basis. Lee et al. [31] used newer cannulated, headless, variable-pitch screws following open reduction. For comminuted fractures, they recommended the use of cerclage wires or sutures. They reported that the compressive force exerted by headless variable-pitch screws allows enough stability to resist proximal migration and rotation at the fracture site. This may explain why their series gave the maximum union rate, greatest functional outcome and lowest complication rate of the reviewed studies.

Unlocked IMN of distal fibular fractures was first reported by McLennan and Ungersma following the development of their Inyo nail made from malleable stainless steel and triflanged to resist torsional stress [21]. However, their initial series gave an unacceptably high complication rate of 16 % due to nail migration and malunion. Following refinement of their technique to include percutaneous clamping and the use of shorter nails, they were able to achieve a reduced complication rate of 10.6 %, with no cases of malunion or nail migration [22]. Pritchett et al. [23] compared rush rods to traditional AO plating methods but only included supination eversion type IV injuries. They experienced an earlier time to weight bearing in the rush rod group (6 vs. 12 weeks) and more complications (deep infection, nonunion, ankle fusion) in the AO plate group. Whilst improved functional outcomes were reported with rush rods, worse radiographic results were seen in terms of fibular shortening, increased medial clear space and posterior displacement. Lee et al. [34] compared the use of Knowles pins to plating and noted that the pin group had significantly smaller wound incisions, a shorter operative time, a shorter hospital stay, less symptomatic hardware and lower complication rates. However, this study was limited by its retrospective nature and nonrandomised group allocation. Importantly, there was no significant difference in functional outcomes at final follow-up.

Advantages of locked IMN include better rotational control, improved stability and reduced risk of nail migration. The first report of locked IMN for distal fibular fractures was published by Kara et al. [26] concerning the ANK nail, which was designed for lateral malleolus fractures with syndesmosis rupture. Whilst all fractures healed, the most significant complication was fibular shortening, which occurred in comminuted, oblique or nonanatomically reduced fractures. Kabukcuoglu et al. [27] reported limited success with the ANK nail, with an overall complication rate of 20.4 %. They correlated a significantly worse clinical and functional outcome with fibular shortening >2 mm. Ramasamy and Sherry [28] provided the first report of a modern fibular nail involving patients with Weber B fractures. However, due to a very small sample size, their results have limited external validity. Rajeev et al. [32] reported on a larger cohort of elderly patients treated with the same implant and type noted that all fractures healed uneventfully with no complications. Functional assessment revealed a mean OMS at 1 year of 58.125, i.e. fair.

Gehr et al. [29] presented the largest study to date regarding IMN of distal fibular fractures. This prospective case series reduces the possibility of recall bias associated with earlier retrospective studies. In addition, a consecutive group of patients was followed, which helps eliminate the risk of recruitment bias within their study population. Whilst acceptable results were reported, two patients suffered severe soft-tissue complications requiring skin grafting procedures. This may have been due to the use of open reduction techniques in some patients with associated complex nonmalleolar distal tibial fractures. Bugler et al. [33] reported radiological and functional outcomes of locked IMN in a large series of patients with 6 years follow-up. An independent and blinded assessor was used to interpret radiographic outcomes, potentially eliminating the risk of investigator bias. However, whilst 76.2 % of patients were available for radiographic follow-up, only 49.5 % responded to the postal questionnaires regarding functional outcome, indicating a significant proportion of their population lost to follow-up. All fractures eventually united, and acceptable functional results were achieved through a variety of validated scoring systems [40, 44, 45]. However, the overall complication rate was 22.8 %. Specifically, complications relating to fixation failure were higher earlier in the series when unstable locking screw configurations were used. Eventually, the combination of syndesmosis and distal locking screws was deemed to be the most stable configuration. Tawari et al. [36] reviewed two matched groups of patients who underwent fixation for unstable Weber B fractures with either IMN or standard AO plating. There was no significant difference between groups in time taken to achieve clinical and radiological union. One patient in the plate group had a wound infection, but there were no wound complications in the nailing group. Most recently, Bugler et al. [37] presented the only prospective randomised controlled trial comparing locked IMN to plating. Whilst 16 % of patients in the plating group developed wound infections, no infections or wound complications occurred in the IMN group. At 1 year, functional outcome favoured the IMN group, but this difference was statistically insignificant. In addition, the overall cost of treatment in the IMN group was lower despite the increased cost of the implant.

Overall, union rates were well reported throughout, with bony consolidation being achieved in 98.9 % (range 88.9–100 %) of patients [21–26, 28–36]. It can be concluded that intramedullary fixation of distal fibular fractures gives excellent union rates comparable with ORIF. However, methods of assessing union were not accurately presented, making this an irreproducible outcome measure. Typically, plain radiographs are used to assess union of long-bone fractures through the presence of callus formation, but this has shown to be unreliable, with wide interobserver variability [46]. In practice, clinical evaluation is essential and incorporates an assessment of pain, tenderness and ability to bear weight; details of how these factors were measured were not presented in the reviewed studies. Functional assessment was undertaken through a variety of assessment tools. Earlier studies used unvalidated patient-reported outcome scores, with good or excellent outcomes reported by the vast majority of patients [21, 22, 25, 26, 30, 31, 35]. The OMS has been used primarily in studies evaluating locked IMN in which 68.8 % achieved good or excellent outcomes [28, 29, 32, 33]. Whilst these results are encouraging, those with unsatisfactory functional outcomes may reflect the natural history of such injuries, which often occur in elderly patients. The OMS was initially conceived to provide a functional assessment tool following an ankle fracture and was tested against subjective evaluation, range of motion, presence of osteoarthritis and severity of initial injury [40]. Whilst widely used, it is important to note that limitations of the score’s validity testing include a relatively small series of patients, inclusion of only bimalleolar fractures and scoring questions relating to running and jumping, which many elderly patients would be unable to do prior to injury. In addition, the presence of syndesmotic injuries with the potential for distal tibiofibular joint instability deserves special mention. None of the intramedullary screw devices [24, 25, 31] and earlier designs of nailing implants [21–23, 26–28, 30, 32, 34, 35] allowed for combined fixation of associated syndesmotic injuries. Therefore, these older devices would not be indicated in more complex injuries, such as high fibular fractures. However, more modern locked nailing implants [29, 33, 36, 37] allow for supplementary syndesmosis screw fixation and are therefore more suited to such injuries.

The mean complication rate across all studies was 10.3 % (range 0–33.3 %) [21–34, 36, 37]. The wide range probably reflects multiple variables, such as different nail design, surgeon experience and complexity of cases. Most commonly, complications involved implant-related problems requiring metalwork removal, failure or fibular shortening [21–37]. The latter being mainly reported with the use of the ANK nail in unsuitable fracture patterns, whilst problems relating to metalwork were associated with either earlier nail design or improper locking screw placement. Interestingly, complications such as mechanical failure were relatively higher in the locked IMN series compared with other studies. Whilst this may appear counterintuitive, it may reflect the learning curve seen in longer studies in which earlier techniques were deemed to be inadequate [33]. The large variation between studies makes it difficult to accurately compare complication rates to standard plating techniques. The most recent studies involving AO techniques show extremely favourable complication rates of 1.7–5 % [47, 48]. However, the only prospective randomised controlled study available for review showed significantly more wound-related complications in patients treated with plates than those treated with IMN [37].

Overall, review of the selected studies revealed that excellent union rates and satisfactory functional outcomes can be expected with intramedullary fixation. However, complication rates can be unacceptably high, although this may reflect a learning curve. Due to the numerous methodological flaws within the reviewed studies, definitive conclusions regarding the clinical application of intramedullary fixation for distal fibular fractures cannot be made. The presence of selection bias, retrospective data collection, lack of control groups and inadequate functional assessment tools provide only poor-quality evidence for fibular nailing. In conjunction with the paucity of high-quality evidence regarding clinical and functional outcomes, practical concerns exist regarding the steep learning curve, expense and appropriate timing of surgery with regards to soft-tissue swelling.

Strengths of this review are the clarity and reproducibility of our search strategy using multiple evidence-based databases. PRISMA guidelines for the reporting of systematic reviews were used throughout in order to increase transparency and reduce the risk of publication bias [20]. Limitations of this review are the inability to pool data for true meta-analysis due to the heterogeneity of individual studies. Also, most reviewed articles were case series, which are prone to both selection and experimental bias. We also acknowledge that although insufficient detail was available in abstracts to allow complete critical appraisal, they were included in our study in order to provide the most up-to-date assessment regarding locked IMN [36, 37].

In conclusion, there is insufficient evidence for changing practice from plating of unstable distal fibular fractures to intramedullary fixation based on the current literature. Adequately powered randomised controlled trials comparing well-matched patient groups with long-term follow-up are required to limit systematic error and enhance external validity. Specific outcome measures should include union, functional assessment, complications and cost–benefit analysis.
